# Are we generating more assessments without added value? Surgical trainees’ perceptions of and receptiveness to cross-specialty assessment

**DOI:** 10.1007/s40037-020-00594-0

**Published:** 2020-06-05

**Authors:** Sarah Burm, Stefanie S. Sebok-Syer, Julie Ann Van Koughnett, Christopher J. Watling

**Affiliations:** 1 Division of Medical Education, Dalhousie University, Halifax, Nova Scotia Canada; 2 Department of Emergency Medicine, Stanford University , Palo Alto, California USA; 3 Divisions of General Surgery and Surgical Oncology, Western University, London, Ontario Canada; 4 Departments of Oncology and Clinical Neurological Sciences, Centre for Education Research and Innovation, Western University, London, Ontario Canada

**Keywords:** Competency-based medical education, Assessment, Postgraduate medical education, Feedback

## Abstract

**Introduction:**

Competency-based medical education (CBME) hinges on robust assessment. However, integrating regular workplace-based assessment within demanding and sometimes chaotic clinical environments remains challenging. Many faculty lack assessment expertise, and some programs lack the infrastructure and faculty numbers to fulfill CBME’s mandate. Recognizing this, we designed and implemented an assessment innovation that trains and deploys a cadre of faculty to assess in specialties outside their own. Specifically, we explored trainees’ perceptions of and receptiveness to this novel assessment approach.

**Methods:**

Within Western University’s Surgical Foundations program, 27 PGY‑1 trainees were formatively assessed by trained non-surgeons on a basic laparoscopic surgical skill. These assessments did not impact trainees’ progression. Four focus groups were conducted to gauge residents’ sentiments about the experience of cross-specialty assessment. Data were then analyzed using a thematic analysis approach.

**Results:**

While a few trainees found the experience motivating, more often trainees questioned the feedback they received and the practicality of this assessment approach to advance their procedural skill acquisition. What trainees wanted were strategies for improvement, not merely an assessment of performance.

**Discussion:**

Trainees’ trepidation at the idea of using outside assessors to meet increased assessment demands appeared grounded in their expectations for assessment. What trainees appeared to desire was a coach—someone who could break their performance into its critical individual components—as opposed to an assessor whose role was limited to scoring their performance. Understanding trainees’ receptivity to new assessment approaches is crucial; otherwise training programs run the risk of generating more assessments without added value.

**Electronic supplementary material:**

The online version of this article (10.1007/s40037-020-00594-0) contains supplementary material, which is available to authorized users.

## Introduction

The field of medicine has undergone transformative curriculum change by embracing competency-based medical education (CBME). This shift is criterion-based and asserts that programs determine a physician’s competence based on a set of pre-defined practice-based outcomes. Implemented effectively, this shift will enable programs to individualize trainee learning, better document their development of competence, and potentially enhance patient care[1, 2]. CBME makes good pedagogic sense, focusing not only on what physicians *know*, but more importantly on what they can *do, *especially in authentic clinical workplaces. However, the success of CBME implementation hinges on robust assessment practices and the creation of an environment conducive to assessment for learning, which presents challenges[3–5].

The clinical environment can be chaotic and unpredictable, raising logistical concerns surrounding the feasibility of introducing flexible and continuous workplace-based assessment into settings where patient care remains the priority[6]. Furthermore, direct observation of trainee performance—a fundamental pillar for accurate and meaningful assessment—occurs infrequently[7–11]. For trainees, engaging faculty to understand their performance data and obtain worthwhile feedback remains challenging. Even when it does occur, faculty lack training in how to judge trainees’ performance and provide detailed, constructive feedback across diverse learning contexts and levels of training[12, 13]. Unlike other domains, the context of medical education is such that faculty are often fulfilling several educational roles (i.e., assessor, supervisor, mentor, and coach) to ensure trainees’ competency develops longitudinally. Each of these roles is distinct in its intentions and goals, yet we often invest in the same individuals to fulfil multiple roles simultaneously, potentially leading to role confusion on the part of faculty and to challenges in the receptivity of feedback by trainees[14, 15].

CBME has the potential to stretch existing assessment resources to a breaking point; training programs need innovative strategies that can both meet assessment demands and motivate trainees to improve their knowledge, skills, and understanding. Introducing assessment strategies that provide faculty with the necessary training and protected time to actually engage in direct observation (i.e. without the distraction of other attending obligations) is essential if we strive to employ assessment processes that are educationally valuable [16–18]. Recent literature has pointed to the need for multiple, dedicated assessors to counterbalance the time and resources necessary to sustain a CBME assessment program[19, 20]. Understanding the need to utilize our existing faculty and resources most effectively, we designed and implemented an approach to assessment that we called “cross-specialty assessment.”[21]. Through this innovation, we demonstrated the potential of training a cadre of faculty from diverse specialties and backgrounds to enact distinct assessor roles around select, procedural and non-procedural skills that transcend specialty boundaries. In our first iteration, MD and PhD faculty were trained to complete formative assessments of trainees’ delivery of patient handover in critical care and pediatric clinical environments. After the assessments were completed, we interviewed faculty regarding their experiences. We demonstrated that faculty could be trained to assess trainees’ delivery of patient handover in specialties outside their domain; however, one of the problems we encountered with real workplace-based assessment was the unpredictability and demanding nature of the clinical learning environment. This left us considering the most appropriate venue to utilize cross-specialty assessment.

Many workplace-based activities that require assessment are unplanned and context specific, raising logistical concerns when wanting to deploy cross-specialty assessors. The simulated environment is more predictable, offering opportunity for observations to occur more consistently, and thus we thought this venue may be a better use of cross-specialty assessors. We know from the surgical education literature that raters can reliably assess those outside their specialty[22] and that non-physicians can play a valuable role in the procedural skills training of trainees[23, 24]; however, trainees’ perceptions of and receptiveness to these creative assessment approaches have not been adequately explored. To address this gap, we wanted to better understand the receptiveness to cross-specialty assessment, specifically from the viewpoint of surgical trainees, a perspective that remains poorly represented when considering how to meaningfully optimize competency-based assessment. If we can better understand how trainees respond when assessments are completed by faculty ‘outsiders’, we can make evidence-informed recommendations about how best to design, share, and implement assessment resources across training programs.

## Methods

The principles of case study research guided the design of this study. Case study research can be an effective methodology for studying a real-world phenomenon within the local context in which it occurred[25]. In this instance, the case was a deep exploration into the perceived effectiveness of establishing distinct assessor roles and then deploying trained assessors to complete formative assessments within a postgraduate surgical training program at Western University. Within the broader study, collected data consisted of multiple sources: field observations of each simulated practice period, semi-structured interviews with assessors, and focus group interviews with trainees. Completed assessments were also collected and examined to provide context for the interviews and focus groups. This paper focuses exclusively on trainees’ perspectives being formatively assessed by outside (e.g. non-surgeon) assessors while performing a basic laparoscopic surgical skill: intracorporeal suturing using a box trainer.

### Setting

All surgical trainees were enrolled in Surgical Foundations, a Royal College of Physicians and Surgeons of Canada (RCPSC) accredited program for first and second year surgical residents. Running parallel to their home training program, Surgical Foundations provides training in surgical fundamentals to residents in a range of surgical disciplines. At Western University, residents from nine different surgical specialties participate in the Surgical Foundations program: General Surgery, Urology, Plastic Surgery, Orthopaedic Surgery, Otolaryngology/Head and Neck Surgery, Neurosurgery, Cardiac Surgery, Vascular Surgery, and Obstetrics and Gynecology. Approximately 30 trainees enter the program each year. An Introduction to Surgery course occurs longitudinally throughout the first year of postgraduate training in Surgical Foundations and is designed to enable trainees to acquire the foundational knowledge and skills of surgery. Led and taught primarily by surgeons (JVK) and senior surgical trainees, this course includes procedural skills training, simulation-based training, and classroom-based lectures. At the end of the program, trainees are required to successfully pass the RCPSC Surgical Foundations exam.

### Study participants

In February and March 2018, we recruited trainees in postgraduate year 1 (PGY-1) of the Surgical Foundations program; all PGY‑1 trainees who were enrolled in the Introduction to Surgery course were eligible for participation. We chose this participant pool for two reasons. First, the initial year of surgical training is when trainees are introduced to the technical skills fundamental to the performance of laparoscopic surgery. Intracorporeal suturing is an essential skill for operative surgery; it transcends specialties and is considered a prerequisite for advanced laparoscopic procedures. Simulation training with accompanying formative assessment is critical for identifying individual trainee strengths and areas for improvement specific to this foundational skill. Second, we intentionally sought to push the limits of our cross-specialty assessors. While we had previously asked cross-specialty assessors to engage with the task of patient handover, for this study we opted to ask them to assess a simulated surgical task. We anticipated that this choice of task would allow us to explore the limits of cross-specialty assessor credibility, and to learn more about the use of this approach for procedural learning.

For this innovation, a total of 27 PGY‑1 surgical trainees were formatively assessed. Eight faculty were recruited to participate as assessors in this innovation; all faculty held appointments in a clinical department at Western University. Six assessors were clinical faculty and two assessors were non-clinical (PhD) faculty. The inclusion of both MD and PhD assessors was intentional; our PhD participants’ previous experience in medical education and recent involvement in CBME initiatives positioned them as potential assessment resources worthy of study. Prior to their deployment, faculty participated in a one-hour training session facilitated by a general surgeon (JVK). This formal training session enabled faculty to become oriented to the materials as well as the cognitive and technical requirements of the task. During training, JVK performed each step of the technique and modelled how she would typically instruct the task to trainees. The task was modeled after the Fundamentals of Laparoscopic Surgery suturing task, which is a validated task set and tool widely used to assess laparoscopic skills in surgeons[26].

Assessors were given time to familiarize themselves with the task, which included time to practice performing the suturing task and to receive immediate feedback on their performance from JVK. Assessors then practiced evaluating their colleagues’ performance of this task for both accuracy and time. The assessment tool for this innovation (see Appendix A of the online Electronic Supplementary Material) was modelled after Chang et al.’s (2016) Objective Structured Assessment of Technical Skills (OSATS) tool[27] and included a procedure specific checklist and open textboxes for assessors to provide narrative comments. Following the training session, faculty were provided with print and video resources enabling them to review the cognitive portion of this task prior to assessing trainees.

Earlier in the Introduction to Surgery course, trainees received focused teaching and supervised practice performing various laparoscopic skills using box trainers. Trainees then had practice time on separate days to practice these skills, and it was during these practice days that cross-specialty assessments took place. Faculty members assessed between 3 and 5 trainees performing the task. Given that many trainees were relatively new to this task, completed assessments carried a formative intent; that is, their aim was to provide trainees with concrete feedback on how to efficiently perform this task without error. A representative of the research team (SB or SSS) was present during each practice period to collect observational data on the interactions between cross-specialty assessors and trainees. Completed assessments were then collected by SB or SSS and copies were subsequently distributed to trainees. All trainees were explicitly informed that these assessments would not count toward their final grade for the Introduction to Surgery course.

## Data collection and analysis

Following the completion of these assessments, SB and SSS facilitated four focus groups to discuss trainees’ experiences being assessed by an outside assessor. Trainees were informed in advance, via the Office of Surgical Education, that during their laparoscopic skills practice time, there would be trained, outside assessors present providing formative feedback on their suturing skills and secondly, that following their practice period, they would be asked to participate in a focus group on the usefulness of the assessments. Specifically, trainees were invited to share their understandings and perspectives concerning the value of this assessment approach and the credibility and feasibility of using outside assessors to provide formative feedback on procedural skills. Compared with individual interviews, conducting focus groups encourages interaction between participants and invites them to engage in honest dialogue with their peers about topics or issues that are perceived relevant to them[28]. SB and SSS were responsible for consenting trainees for each of the four focus groups. During the consent process, the purpose and study design was clearly described as well as how each trainee’s identity would be safeguarded. Trainees were informed that their participation was voluntary and that they had the right to withdraw from the study without consequence. In total, 27 trainees participated in the focus groups with 6–8 trainees in each group. Focus group questions included: What factors are important for high quality assessment and feedback? What constitutes a credible assessor? How will the feedback you received support your future practice? And what aspects of your performance would be reasonable for a trained assessor outside your specialty to assess? (see Appendix B of the online Electronic Supplementary Material). Each focus group was 30–60 minutes in length, audio-recorded, and transcribed verbatim. Each participant was assigned a unique identifier during transcription to ensure their anonymity.

We then conducted a thematic analysis of trainees’ focus group interview data. We took an inductive approach to our process of analysis, meaning the themes identified within the focus group interviews were strongly rooted to the data themselves[29]. Specifically, we drew on Braun and Clarke’s (2006) six-phase guide to performing thematic analysis[30]. First, SB and SSS individually engaged in a process of ‘repeated reading’ of the data in search of meaning and patterns. At the same time, SB and SSS engaged deliberately in the act of memoing to record early analytical insights and mark ideas for coding that would be returned to in subsequent analytic phases. SB and SSS then met in person to create a coding manual that included an initial set of codes derived from participants’ experiences. Through a process of constant comparison, SB coded the entire data set using NVivo10, a document and coding management software, giving full attention to each data item while continuing to record insights and meaning patterns. CW participated in team meetings throughout the data analysis process to review and refine themes related to our research purpose. During these meetings, we discussed the similarities and differences among resident perspectives and, later, began to identify the overarching ‘story’ each of the different themes revealed, asking questions such as: ‘What assumptions underpin each theme?’ ‘What are the implications of this theme?’ ‘What conditions are likely to have given rise to it ’[30]? Researcher reflexivity was maintained during all steps of the research process through ongoing note-taking and research team discussions.

Our research team included: SB, who is both a PhD trained researcher and educator, committed to improving the trainee experience in the clinical workplace; SSS, who is a PhD-trained researcher specialized in measurement, assessment, and evaluation; JVK, who is a general surgeon with a MEd in Health Professions Education, and CW, who is a non-surgical physician and a PhD-trained researcher focused in feedback. The distinct expertise and professional experiences of each research team member allowed for a broad and critical reading of the data and an increased understanding of the phenomena under study.

Ethics approval for this study was obtained by The Office of Human Research Ethics at Western University (file number: 108391).

## Results

Trainees’ perceptions of having outside assessors formatively assess a basic surgical task such as laparoscopic suturing varied. A few trainees found the experience motivating and expressed satisfaction in the quality and detail of feedback provided. More commonly, however, trainees questioned both the feedback they received and the practicality of cross-specialty assessment in furthering their professional growth, at least in the realm of procedural skill acquisition. We begin this section by outlining trainees’ initial perceptions of our assessment innovation, specifically their views on having outside assessors’ complete formative assessments around a procedural task. We then shift to discussing what constitutes meaningful feedback and trainees’ perceptions around the effectiveness of separating assessment from feedback.

### Trainees perceptions of cross-specialty assessment

Trainees experiences of being directly observed by outside assessors varied. While relatively new to laparoscopic suturing, some found it useful having assessors present to explain “all the steps that were required to do the procedure completely” (R3, FG 4). Having their skills and behaviors observed by an assessor outside of surgery encouraged trainees to “pick up the pace and try a little harder” (R1, FG 2). Others felt the experience created an environment where trainees felt they needed to “perform as you would on an examination” (R2, FG 2).

Frequently, however, trainees found themselves questioning the educational value of cross-specialty assessment. Accustomed to receiving immediate feedback within the clinical settings of their surgical programs, some trainees appeared ambivalent about the purpose and use of these assessments, particularly in a surgical program where a trainee’s performance is often influenced by the performance of his/her team members. As one trainee explained:

*I’m a junior resident, so I’m always being supervised in some way … your fellow or whoever is right by your shoulder. I honestly can’t think of a time when I’m not being either directly or indirectly supervised where I would require another person to assess me*. *(R1, FG 4)*

Others appeared somewhat disappointed with the outcomes of their assessment: “Everything I received feedback on was stuff I had heard in the past and knew I was doing wrong” (R5, FG 1). Quite often, trainees described the observational feedback they received as vague or generic; “there’s no expert opinion” (R2, FG 3), leaving some trainees desiring more:

*It was better than having no assessors because you had an objective person critiquing your technique… But not as good as having someone … who knows you, who can critique on your whole learning, where you are in your learning … and what you can do to move forward … the impact didn’t last as much as it does when I get feedback from someone I know*. *(R4, FG 2)*

Assessors appeared capable of “ticking the boxes” (R6, FG 2) and communicating procedural errors, but what trainees sought went beyond mere observation of their performance; they wanted assessors to identify strategies for improvement. As one trainee remarked, “I just don’t see the point of getting feedback if it’s just for the sake of filling out a form and not for the sake of improving your future practice” (R1, FG 3).

### Credible and constructive feedback

Trainees desired feedback that was both credible (feedback from someone they can trust) and constructive (feedback they can use to enhance professional growth). Trainees sought feedback from individuals with accumulated experience and know-how; “someone with more skill than me in the task that’s being assessed” (R1, FG 3) and who “can do a great job explaining how to do that task better” (R3, FG 2). Additionally, they wanted feedback for the purpose of continuous improvement:

*For things that I am going to be using in my practice one day, for things that I’m going to need to be able to build on in order to get higher-level skills later on, I think that the person should at least be able to do the skills themselves so that they can provide meaningful feedback to you about how to improve your technique (R1, FG 4)*.

It is not surprising then, that some trainees questioned whether outside assessors were capable of providing high-quality feedback around a task such as laparoscopic suturing: “I could not possibly fathom anyone not in my specialty evaluating me on skills that are very much context dependent … you draw from their expertise and that’s what actually improves you.” (R5, FG 3).

Standalone assessments without valuable feedback were perceived by trainees as “useless” (R1, FG 1). As one resident remarked, “You can’t boil everything down to a checklist … there’s some error in trying to evaluate a non-binary skill using a binary checklist” (R6, FG 3).

What trainees appeared to desire was real-time coaching; someone who could break their performance into its critical individual components. Recalling the feedback one trainee received from a staff surgeon: “She [the surgeon] could physically show me and take my hands and move me through the motion. I think that’s a huge benefit that I don’t know you would necessarily get from someone who doesn’t do that every day” (R2, FG 2). Specialists were seen being able to “provide real feedback, technical feedback” whereas outside assessors were described as providing “general feedback of how to score better” (R4, FG 2) on the task checklist.

### Fostering the feedback alliance

Trainees appeared uncomfortable having outside assessors merely judge their performance. Rather, they wanted someone who could see their areas of weakness as opportunities for improvement. We wondered, then, how pre-existing relationships influenced the quality of the feedback delivered during formative assessments. We discovered that trainees did not necessarily care who completed assessments *for* learning, so long as the person provided what they perceived as credible and constructive feedback. Many saw the relationship between assessor and trainee as transactional: “I’m not trying to become his best friend during the session. I’m doing a skill and I’m expecting something in return. It doesn’t have to be somebody who I know” (R2, FG 3). While some did not necessarily need to have a longitudinal relationship with the person assessing them, they did need to know that the person had domain expertise: “You need someone who’s done it thousands of times, way more than you have, and [provides] the tips and tricks they have in terms of doing this specific procedure … that’s where you often get that good feedback” (R7, FG 1). For others, having established relationships with the person completing the assessment made it easier for “internalizing the feedback” (R2, FG 1). As this trainee further explained:

*You need someone who is invested in your personal residency program … I need somebody who chose me to be there and is invested in me to complete this … you need someone that’s going to be like, this is what you are doing wrong and how can I help you change this? (R2, FG 1)*

Longitudinal relationships between trainees and faculty seemed to contribute to the provision and acceptance of feedback but appeared less critical for the assessment component of the task. Trainees responses highlighted the importance of trust—trust in the assessor’s familiarity with and ability to perform and teach the task as well as trust in the specificity and substance of the feedback delivered.

### Credibility in the eyes of trainees

It was important during the focus groups to explain to trainees how and why an assessment intervention like this was developed. Nevertheless, trainees’ expressed much trepidation at the idea of using outside assessors to meet increased assessment demands: “The idea of intentionally giving [outsiders] the power to assess trainees … is a very scary thought … there needs to be a very important gate-keeping role in determining who gets to make these evaluations” (R4, FG 1).

Many wondered what might be lost, expressing concern that increases in assessment data does not necessarily equate to better trainee development:

*Nothing is black and white with medicine, everyone has different techniques for the same procedure … if people from outside your specialty come in with a special rubric in mind, and then you’re trying to meet those rubrics, then you lose really what you perceive as good medicine*.* (R6, FG 2)*

Particularly in surgery, trainees felt structured assessments, like the ones conducted in this innovation, did not fully capture all of what it means to be a competent surgeon. Trainees looked to specialists not only to facilitate improvement in their procedural skills but also to learn “how to make decisions and how to think” (R2, FG 2) like a surgeon. Honing that tacit (‘know-how’) knowledge was important to trainees and thus the feedback they received needed to come from someone with “in-specialty knowledge” (R4, FG 1).

Throughout the four focus groups, trainees alluded to the fact that there might be a contextual threshold for cross-specialty assessments. Trainees were reluctant to embrace the notion that procedural skills, even in a program such as Surgical Foundations where basic skills are taught, could be appropriately assessed by outsiders. As one resident from general surgery remarked:

*If I look at our three biggest bread and butter procedures—hernias, gallbladders, and appendixes—they can actually be very technically difficult … I just can’t see an outsider, even if they’ve seen a few gallbladders, be able to sit there and complete checklists … I just don’t know how that would benefit me doing the procedure as a whole*. *(R4, FG 1)*

A few trainees suggested this assessment strategy might be better suited to trainees in medical school versus residency. However, even at that level of training, some felt you would be losing “the experience and expertise of the actual people that do this as a specialty” (R6, FG 3). As one trainee pointed out: “Someone evaluating the movement of your wrist, that’s very much something that comes from experience … you can’t put that in a checklist … non-specialized people, they just can’t give you that instruction” (R5, FG 3). Trainees perceived the use of a checklist tool impeding outside assessors’ ability to provide authentic feedback. Although the checklist enabled assessors to record and confirm completion of each specific step in the task, it failed to adequately capture the nuance of the task, which many participants felt inhibited rich, reflective feedback conversations from occurring between trainees and assessors.

## Discussion

We piloted cross-specialty assessment to address a pragmatic concern generating international dialogue in recent years: the potentially overwhelming assessment demands of CBME implementation on clinician educators. We formally trained a cadre of dedicated non-surgical physicians and non-clinical faculty to complete formative assessments around a basic surgical skill because we perceived it as a line of defense to optimize available resources and alleviate assessment fatigue among surgical faculty. However, surgical trainees’ responses to this novel assessment approach raised concerns. While a few trainees spoke positively about their experience, many trainees questioned the feedback they received and the practicality of this assessment approach in advancing their procedural skill acquisition.

Often, trainees perceived receiving a score rather than valuable feedback from assessors, a finding that is consistent with what others have reported concerning trainees’ experience of assessment and feedback[4, 31, 32]. Trainees’ apprehension with the idea of using outside assessors appeared rooted in two areas: skepticism around assessor skill level and a perceived loss of nuance in feedback they received regarding their performance. Within our study’s context, trainees wished for their clinical teachers to fulfill both the assessment and the coaching role simultaneously; in other words, they sought interactions with individuals who could assess their progress meaningfully *and* provide trustworthy feedback that would help them improve. These findings tell us that accurate assessments alone, without accompanying credible feedback, are viewed as sorely lacking in value for learning.

Our study is the first we know to empirically explore the impact of training a group of faculty members to enact defined assessment roles. We approached this task with confidence that with formal training, our faculty would be able to complete accurate and informative assessments specific to the observed procedural task. Nonetheless, we wondered whether accurate assessments would prove sufficient, and whether trainees would perceive the exercise as valuable. Interestingly, trainees’ reaction to our assessment innovation revealed what proponents of CBME might hope—that trainees approach competency-based assessments as opportunities to meaningfully engage in, reflect upon, and monitor their learning and development. Perhaps less expected was trainees’ thoughtfully articulated explanation around what is lost when assessments are perceived to confine trainees to the “tasks of doctoring” versus opportunities to engage in “learning to ‘be’ a doctor” [33].

The value of this paper lies in its transparency and candor. What is often absent from studies reporting the impact of a new educational innovation is a critical examination into what obstacles arose and inevitably needed overcoming [34]. We deliberately chose to outline the educational decisions that were made throughout the design and implementation of cross-specialty assessment; some of these were informed by the theoretical tenets and design principles of programmatic assessment [4, 20, 35, 36], while others were made to purposely address the logistical and administrative challenges embedded in medicine’s assessment culture [37, 38]. More importantly, however, our contribution lies in what can be learned when an assessment intervention moves from meticulous planning and preparation into the reality of the clinical training environment. Certainly, there are times when assessing and not providing feedback is appropriate (e.g., during high-stakes licensure and certification examinations). However, from what trainees shared, it appears these instances are uncommon, and in routine learning situations, assessment without feedback appears distinctly undesirable. We do not claim to have figured out the optimum balance between assessment and coaching. Rather, we believe our findings highlight continued points of tension and serve as an exemplar for determining the appropriate balance of coaching and assessing in particular contexts. The findings from this study add an additional layer of complication for those advocating for the separation of coach and assessor roles. Perhaps it is overly simplistic to think we can separate the two roles; the findings from this study prompt us to shift our focus to thinking about how we can develop clinicians to concurrently take on the role of assessor and coach without diminishing their ability to be effective.

Is there any way forward for cross-specialty assessment? We have asked ourselves this question, wondering whether this assessment approach is worth the investment of time and resources required for long-term sustainability. In our previous study [21], we learned that completing workplace-based assessments in the clinical environment is logistically complicated because of the unpredictable nature of clinical work; additionally, an incredible amount of faculty training is required even to assess a simple task. In this study, we learned we can train outside assessors, but even when you alleviate some of the logistical concerns and put assessors into a seemingly predictable venue where direct observation can occur uninterrupted, it cannot be assumed that assessments intended to drive learning will, in fact, yield change in knowledge or behavior [39].

If future iterations of cross-specialty assessment are to be pursued to better support formative assessment, our recommendation is it be used to formatively assess non-technical skills that really cross specialty boundaries (e.g. breaking bad news, informed consent) or as a form of pre-assessment as trainees transition from medical school to residency. Cross-specialty assessors might also be effectively deployed in summative circumstances, where the requirement is for assessment more than feedback. We caution training programs to carefully consider the increased administrative requirements and needed faculty development before implementing this assessment approach, especially if the situation calls for formative assessment practices. We should not underestimate trainees’ expectation for feedback; trainees can only act to improve if the information provided to them is useful and trustworthy.

Our study is limited by some of the design decisions we made during the conceptualization and implementation phases of this innovation. First, faculty training for this innovation was brief. We aspired to introduce an assessment process that showed potential for sustainability and thus wanted to ensure a feasible investment in training. Second, to maintain transparency and reiterate the formative intention of cross-specialty assessment, all trainees were informed that assessors were not surgeons, but had been formally trained to assess their laparoscopic suturing skills and to the extent that they felt they could provide narrative feedback. Perhaps because trainees knew assessors were not surgeons, their expectations for meaningful coaching may have been low at the outset and therefore they viewed assessors as simply offering a judgement. Because surgical training features extended moments of feedback and direct observation in the operating room around real patient cases, it may also be that trainees in this domain had particularly high expectations for feedback. We did not directly compare the value of practicing the surgical task without any assessment or feedback (solo practice) to practicing and receiving feedback from a cross-specialty assessor. Finally, we acknowledge that we made no effort to confirm or demonstrate inter-rater reliability between assessors; rather, our focus was on trainees’ receptivity to cross-specialty assessment. Accuracy may be irrelevant if trainees perceive the whole exercise as not very useful.

## Conclusion

The move towards CBME has prompted training programs to think creatively about how to enhance assessment processes that can equally support trainee growth and monitor competency progression. Recognizing the time constraints faculty are faced with, we introduced cross-specialty assessment, an approach designed to deploy trained faculty to formatively assess trainees in specialties outside their own. The introduction of cross-specialty assessment, while a good idea in principle, did not appear an effective strategy for mitigating the potential assessment burden of CBME, nor was it received favorably by trainees, leaving us thinking more about what is lost when you attempt to separate the assessor from the coach. Understanding trainees’ receptivity to new assessment approaches is crucial; otherwise training programs run the risk of generating more assessments without added trainee value, compromising assessment quality in favour of assessment volume.

## Caption Electronic Supplementary Material

1. Appendix A. The assessment tool for this innovation and the interview guide used during focus groups with trainees can be found as ESM

2. Appendix B
