# Supplementary material for: Are we generating more assessments without added value? Surgical trainees’ perceptions of and receptiveness to cross-specialty assessment
Source: Perspect Med Educ. 2020 Jun 5;9(4):201–9. doi: 10.1007/s40037-020-00594-0 (PMC7459015; doi:10.1007/s40037-020-00594-0)
Supplement: Supplementary file 1 — 1. Appendix A. The assessment tool for this innovation and the interview guide used during focus groups with trainees can be found as ESM [file 40037_2020_594_MOESM1_ESM.docx]

**Cross-specialty Assessment Codebook –Surgical Foundations**

**Residents’ perceptions of assessors’ credibility:** Use code when residents describe an action or indicator that led them to perceive assessors as credible or not.

**Can we vs. should we:** Use this code when participants discuss the benefits and drawbacks of this assessment approach. This could include logistical, feasibility and sustainability challenges.

**Defining credibility:** Use this code when participants share their understanding of what credibility is. Also apply this code when participants describe factors that according to them, favorably contribute to or impede credibility.

**Formative Assessment:** Use this code when residents describe the assessment data they received. This code can be applied to capture *what specifically they received feedback on*

- **Coaching:** Use this code when residents describe an element of teaching, coaching, mentoring, or other that would lead to improvement in practice or development in skill.
- **Feedback:** Use this code when residents talk about discussions, limitations, and/or perspectives on both the feedback they received or wished they received
- **Purpose of assessment:** This code can be applied when residents describe what they felt was the purpose of having a cross-specialty assessor and receiving assessments in this context.
- **Perceived quality of assessment data:** Use this code when residents list factors that strengthened or compromised the quality of assessment data they received. Also use this code when residents describe circumstances related to the quality of the assessment they received.

**Miscellaneous:** Use this code for excerpts that standout but don’t necessarily fit within existing codes.

**Opportunities for cross-specialty assessment**: Use this code when residents describe other tasks where a CCA approach might work or not.

**Recognizing limitations:** Use this code when residents acknowledge potential limitations of the assessment tool or assessment experience.

**Washback**: Use this code when residents describe some impact that the assessment or innovation has had on their learning. Examples can be positive or negative and can either maintain or hinder educational goals. (something that wasn’t there before)
