# Supplementary material for: Are we generating more assessments without added value? Surgical trainees’ perceptions of and receptiveness to cross-specialty assessment
Source: Perspect Med Educ. 2020 Jun 5;9(4):201–9. doi: 10.1007/s40037-020-00594-0 (PMC7459015; doi:10.1007/s40037-020-00594-0)
Supplement: Supplementary file 2 — 2. Appendix B [file 40037_2020_594_MOESM2_ESM.doc]

**Can we vs. Should we**

R6: Even that, though. I feel like the benefit of feedback is, coming from someone who has done that and has had those same struggles and maybe understands why.

I think the problem, too, is now we’re narrowing our scope so significantly. So, when you’re in medical school, you can have a family doctor watch you do a history and physical because you’re being all inclusive. But now, for urology, I don’t really want a family doctor watching who’s going to be like, oh, you didn’t ask about this aspect of family history. I want my program director, one of my staff, who’s going to say, you didn’t need to ask about this. You could shave off time by doing this, this, this, and this. And you would only know that if you were living that specialty. So, you still could do it for that, but even that I think as we’re trying to become more expert, that it becomes less helpful.

R4: This is Name-X. I’d say that it makes you less nervous to have somebody there that’s an outsider, because this isn’t somebody that you’re going to be working with again. And I find sometimes when you have someone assessing you who you work with frequently, you really, really want to be impressive. And whenever they give you feedback sometimes you take it a little bit more personally and you’re worried that they’re going to treat you differently the next time. But, if it’s somebody that you’re never going to work with again, it’s less nerve-racking I guess you could say.

R2: Sure, yeah sorry, with my mouth full. I think, also to kind of pick up where R1 was, what she was saying, I think it can be … I was only assessed by the other surgeon, I didn’t have the other assessor there. But, I think it could be helpful to have the outside assessor for a couple of reasons. One is, even just getting the feedback at the end with the step-by-step evaluation was useful, and I think anybody should be able to be trained to do that. And then the other thing is I think if whether or not the assessor is a practising surgeon doesn’t necessarily matter, but I think they should have enough knowledge to be able to do the task themselves, have enough experience to be able to do the task themselves so that they can provide feedback that’s more specific to …

I just wanted to clarify. The role of the assessor, is it to just evaluate someone, or is it more to be able to teach? Because I think R1 mentioned earlier, to be able to skilled enough to not only assess but also teach the task ends up being a full-time position. And that may be too resource intensive to have that. So, if you’re looking for the assessors to just evaluate someone, and just playing the devil’s advocate, it might even be better if they’re just trying to … they just have this simple checklist that they’re just following through saying yes or no. But then you should also have some teachers who can look at it after and actually tell you how to improve on that.

R3: I wasn’t saying that you shouldn’t definitely get some feedback so you can improve on it. But there needs to be someone who can just basically assess a whole bunch of things. What I was trying to say is not everyone needs to be able to say, you do 10 of these, let’s say, and they look at your three best and say, hey, these are the things that you commonly make mistakes on. You can get one person who can tell you, you know, I’m thinking of, if you think of you’re doing a whole bunch and there’s some common mistakes that you make. If an assessor who is just purely evaluating you points out and says, hey, you’re using your right hand too much, you’re using your dominant hand too much …

R1: I recognise that, but I don’t feel like receiving feedback without receiving the how I improve on the feedback is going to be helpful. Because I have a limited number of hours in the day. None of us work a 9:00 to 5:00 job, we work a 6:00 to 8:00, 10:00 job. So, adding an extra evaluation in there that’s not helpful to me is not beneficial. Does that make sense? Taking a half hour of my day to go through that task and be evaluated on it but not actually learn something from it is quite honestly a waste of time.

**Coaching**

R4: I’m giving examples, I think there needs to be an important gate-keeping role in determining who gets to make these evaluations because it’s always been decided by the faculty, right or wrong, but it’s about careful selection and making sure that there’s no bias and these are objective measures because if they’re subjective and they just don’t like the way you do something however, despite you being able to do it well and in any other system you would be passed but you’re failing in this system, I think that’s an issue.

I think you need a little bit of subjectivity and you need somebody who’s invested in your personal residency program. You can’t have an outsider because I need somebody who chose me to be there and is invested in me to complete this otherwise you’re not going to get that feedback, you’re not going to learn, you’re going to get x, x, x, whatever, and you’re not going to meet your EPAs, you won’t graduate. You need somebody that’s going to be like, this is what you’re doing wrong and how can I help you change this.

R2: I second that opinion. I would second that opinion. Even today, there was one thing I was struggling with, when my suture got really short, and that’s not a question that I felt anyone else was able to answer. But Dr. X was able … oh, yeah, if you do this, and just change this little bit of positioning of your hand. Not even just to tell me that, if someone else had read about these kinds of complications you could run into and how to address them. She could physically show me, and take my hands and move me through the motion. I think that’s a huge benefit that I don’t know that you would necessarily get from someone who doesn’t do that every day.

R2: No, it doesn’t matter for assessment. But in terms of your learning, I think it’s a lot easier. Dr. X is very invested in all of us, so for her, she doesn’t care about us making the time and just passing. Where an assessor, that might be their only goal is to make sure that we make this milestone and we’re able to pass the test. But for her, it’s like, that we become competent surgeons, and that we are good at what we do. Whereas, they’d be like, okay, well, you did this, and you got it in the time limit, awesome, you didn’t screw up. But she would look at it as, oh, but if you do this, you could be more efficient. If you did that, you could be … you would be in better ergonomic position. Those kinds of little things that would help you in your career, that I don’t think necessarily matter on an assessment.

R4: I think it was better than having no assessors because you had an objective person critiquing your technique and what you’re doing, and what steps you’re missing. But it’s not as good as having someone you’ve asked, who knows you, who can critique on your whole learning, and where you are in your learning, and what you did better, and what you can do to move forward. Then when you have that feedback from someone you know, you’re more invested in it and you think about it. Because in medical school, we had these assessments all the time for OSCEs, and I remember I just got my mark, and I never looked at that page again because it didn’t really matter. It was a one-time thing, they didn’t … it was the score that mattered, and if you passed, you passed. It wasn’t someone I knew, who I was invested in learning from. I think when I reflect back on that, which is very similar to what it was like today, it is useful because you have someone who tells you what you’re doing wrong, and then what you can improve on. But honestly, it didn’t … the impact didn’t last as much as it does when I get feedback from someone I know.

R4: It’s like, if they can’t offer something for you to improve on, as we’re all learning, even once you finish residency and you’re in practice, you’d still want someone who is able to, what would the next step be here, what’s something you can improve on.

R2: It would depend on what you were communicating. If you’re consenting someone for surgery, probably go with the surgeon. If it’s breaking bad news, I’m sure anyone could probably do that who is a physician. If it’s discussing family planning, probably wouldn’t use an ortho-paed to do that, I’d probably go with … Yeah, I just feel like yeah, obviously that’s pretty self-explanatory that certain people are better at … To further on the other student’s comment, I think it’s also important to know what type of training people have had, too. Did they watch a YouTube video, were they just given a rubric and shown a video of what’s considered perfect? The other part of that is, how well they’re trained will dictate how much feedback they can give you that’s useful, which I think someone else talked about, too. If you say, oh, good work, excellent, you made all the tick boxes, it’s like, okay, but I want to improve, that’s why I’m here. That’s why I’m training, I want to get better. What can you offer me in that regard? I think it’s important to make that separation between assessments, and how an outside assessor can help you to achieve the goal of passing the upcoming exam. Sure, that’s useful, versus your actual education. I think sometimes we as learners need to also separate those two things, too, to see the value in each.

R2: They’re teaching you how to make decisions, and how to think.

Part of the problem too, I think is, we might actually have more experience than some of the people who are watching us too, right? Like, R4 has seen how many gallbladders? We’ve closed skin. We’ve done all those things. So, to have someone who has just watched a checklist comment on things that we need that next level is maybe not as helpful. So, even if you had residents who were above you, that might be more beneficial. As adult learners, like R2 said, we know when we’ve hit a wall that we are not good at it. We don’t need to be shown that we are not good at it, we need to be shown how to fix it.

R2: What I’m trying to say, if a soccer player cannot teach somebody how to become an artist, even if he has in front of him the tools he should use, the amount of ink he should use, there’s this feeling, this artistic feeling, that you cannot appreciate unless you are this artist. And the same for surgery, it’s way beyond steps and (inaudible). It’s the sense of surgery. And I can give you a realistic example that happened a couple weeks ago where I knew that the next day, I was on anaesthesia rotation and I had to put in a central line. And I knew the chapters by heart. I went, oh, it’s going to be easy. I memorised these 20 steps. Next day the anaesthesiologist is going to be amazed. No. And I said no, I’m following the steps. Where’s the mistake? What am I doing wrong? He came to me and he’s like, ah, you’re right. You’re going at a 40-degree angle, but try to hold your needle when you go in, up. And this wasn’t in nowhere, in any of the protocols. So, this expertise that he had changed that entire thing. And from that point was like, oh, this thing is amazing.

R4: For example, Dr. X came and I asked her to show me something. Because I’ve seen her do laparoscopic work and I know how good she is, so I know she can help me improve. I think there’s even more merit to that than you know somebody’s a staff but let’s say you’ve never worked with them, so you don’t know if they’re good or not. You can still take their advice, but a lot of times I’ll even check with another person I’m comfortable with and say, hey, this person taught me this. Is that good? And they’ll be like, no, I wouldn’t do it that way, they might have gotten used to that. So, I think there’s kind of a hierarchy that goes with it. But for sure, like R1 said, somebody who’s definitely higher than you. And even senior residents are great at teaching, especially if you’ve worked with them and you know that they’re competent on their own.

R4: Well, I think with CBME it’s supposed to be all about feedback and trying to slowly develop skills, and then not only develop them but get better at it to be a competent surgeon in the end. And so, I think that assessing people, so often in CMBE I think you don’t … not fail, but get quoted as in progress versus complete. And so, if someone is assessing you and it’s going to say this is in progress, they need to be able to tell you how to get from in progress to completed. And to me, teaching and assessing is different, but I feel like you really should be able to teach a task to be able to assess it too. I just feel like that would be a way to know that you’re credible in being able to assess it.

**Feedback**

R2: You need somebody who’s done it thousands of times, way more than you have and these are the tips and tricks they have in terms of doing this specific procedure and that’s where you often get that good feedback.

R2: I think with the assessments, if it doesn’t generate feedback, even if I’m told I’m competent, it’s not valuable. If the goal is to get these EPAs and get a certain number to say you’re competent in that skill and it’s not by the expert and it’s by an independent assessor and you don’t get feedback, specialty feedback, from the independent assessor, how are you going continue to prove to get to the expert level just by achieving competency, it’s not valuable. Because even if I’m … if I pass these tests based on the checkmarks or the rubrics, it doesn’t make a good laparoscopic suturer, it just means that I’ve achieved a certain score as based by an independent assessor, it doesn’t mean I’ll be good in the operating room.

R1: I think you need a little bit of subjectivity and you need somebody who’s invested in your personal residency program. You can’t have an outsider because I need somebody who chose me to be there and is invested in me to complete this otherwise you’re not going to get that feedback, you’re not going to learn, you’re going to get x, x, x, whatever, and you’re not going to meet your EPAs, you won’t graduate. You need somebody that’s going to be like, this is what you’re doing wrong and how can I help you change this.

R1: You need a coach.

R2: That’s what you need, that’s what residency is. All your staff are … every staff I work with is invested in making me a better obstetrician gynecologist because they’re graduating me and I’m representing this school. So, if you have these outsiders that can just be like, no, they didn’t get the circle, the dot, then that I think can be very dangerous.

R2: Even today, there was one thing I was struggling with, when my suture got really short, and that’s not a question that I felt anyone else was able to answer. But Dr. X was able … oh, yeah, if you do this, and just change this little bit of positioning of your hand. Not even just to tell me that, if someone else had read about these kinds of complications you could run into and how to address them. She could physically show me, and take my hands and move me through the motion. I think that’s a huge benefit that I don’t know that you would necessarily get from someone who doesn’t do that every day.

R4: I also agree that was the major down side. They can provide you objective feedback based on their training, but that’s what we get from our textbooks, too. Dr. X was able to provide honestly real feedback, technical feedback. Whereas, the assessors just gave me very general feedback of how to score better. It is, I’d say that is the major down side of having a non-specialty person assess your skills.

R!: I think a yes or no checklist is very black and white. And when you’re actually performing a physical task which is actually most of CBME. In terms of each unit is … this is the skill that we want you to have in that there’s … needs to be able to take a history, needs to know all the knowledge, all that. But there’s usually some sort of physical task associated, or many times. And in order to be able to assess that, a yes or no checklist, and then not giving you feedback on how to improve, I don’t think that’s useful to me. It’s like, oh yes, you were able to thread the needle through the dots, but no, you were not able to do the two ties. Okay, if you think about it that way, and that’s all you get, you’re like, well why wasn’t I able to do the two ties? What am I doing wrong that makes me not able to get this checkmark? And if the person who’s watching you and giving you the yes or no isn’t able to tell you that, I think that it’s kind of useless.

**Recognizing Limitations**

R4: I guess that can create some issues if you’re getting the wrong feedback. If you’re being given feedback by someone that’s not inside the specialty, they can be giving you information or feedback on your technique that’s incorrect and then you go and you perform that same task in front of someone from inside and then you were taught incorrectly or corrected incorrectly and then you’d be doubly incorrect.

So, is the goal of this to investigate whether it would be appropriate for outside assessors to evaluate us on potential EPAs and things like that, and CBE? So, my concern about the issue with that is that the CBME format is designed so that residents can initiate feedback from each competencies but also design situations where they can create formal feedback sessions, not just, you did great on this rotation, go home and read more. It’s, how was my suturing, how was this? So, having an outside assessor on that I think can be quite challenging when you’re … I know some of the urology EPAs are assessing a patient with septic stone, we’re not going to have an outside assessor for that, it doesn’t make any sense. Or if you can put a nail in a hip, it doesn’t really make sense for an outside assessor to do that because you need in-specialty knowledge. So, my question is, what areas can we apply this in? I guess that’s your research question too.

R1: I don’t think that … I mean, this has nothing to do with your research project or anything like that, but the EPAs don’t really facilitate residency. Residency is happening and the EPAs may or may not happen, they’re two different things. Assessments without valuable feedback, again, are just useless.

M: I think the Royal College would say that EPAs are happening.

R1: Yeah, I know, but I mean, they’re separate things from the normal daily

R2: I second that opinion. I would second that opinion. Even today, there was one thing I was struggling with, when my suture got really short, and that’s not a question that I felt anyone else was able to answer. But Dr. X was able … oh, yeah, if you do this, and just change this little bit of positioning of your hand. Not even just to tell me that, if someone else had read about these kinds of complications you could run into and how to address them. She could physically show me, and take my hands and move me through the motion. I think that’s a huge benefit that I don’t know that you would necessarily get from someone who doesn’t do that every day.

R6: Part of the problem too, I think is, we might actually have more experience than some of the people who are watching us too, right? Like, R4 has seen how many gallbladders? We’ve closed skin. We’ve done all those things. So, to have someone who has just watched a checklist comment on things that we need that next level is maybe not as helpful. So, even if you had residents who were above you, that might be more beneficial. As adult learners, like R2 said, we know when we’ve hit a wall that we are not good at it. We don’t need to be shown that we are not good at it, we need to be shown how to fix it.

R1: I think the problem, too, is now we’re narrowing our scope so significantly. So, when you’re in medical school, you can have a family doctor watch you do a history and physical because you’re being all inclusive. But now, for urology, I don’t really want a family doctor watching who’s going to be like, oh, you didn’t ask about this aspect of family history. I want my program director, one of my staff, who’s going to say, you didn’t need to ask about this. You could shave off time by doing this, this, this, and this. And you would only know that if you were living that specialty. So, you still could do it for that, but even that I think as we’re trying to become more expert, that it becomes less helpful.
